# Bio-Cementation in Construction Materials: A Review

**DOI:** 10.3390/ma14092175

**Published:** 2021-04-23

**Authors:** Dawood Muhammad Iqbal, Leong Sing Wong, Sih Ying Kong

**Affiliations:** 1 College of Graduate Studies, Universiti Tenaga Nasional, Kajang 43000, Malaysia; dawood.iqbal@uniten.edu.my; 2 School of Engineering, Monash University Malaysia, Bandar Sunway 47500, Malaysia; kong.sih.ying@monash.edu

**Keywords:** carbon emissions, microbiologically induced calcium carbonate precipitation, urea hydrolysis, binders, construction materials

## Abstract

The rapid development of the construction sector has led to massive use of raw construction materials, which are at risk of exhaustion. The problem is aggravated by the high demand for cement as binding powder and the mass production of clay bricks for construction purposes. This scenario has led to high energy consumption and carbon emissions in their production. In this regard, bio-cementation is considered a green solution to building construction, because this technology is environmentally friendly and capable of reducing carbon emissions, thus slowing the global warming rate. Most of the previously published articles have focused on microbiologically induced calcium carbonate precipitation (MICP), with the mechanism of bio-cementation related to the occurrence of urea hydrolysis as a result of the urease enzymatic activity by the microbes that yielded ammonium and carbonate ions. These ions would then react with calcium ions under favorable conditions to precipitate calcium carbonate. MICP was investigated for crack repair and the surface treatment of various types of construction materials. Research on MICP for the production of binders in construction materials has become a recent trend in construction engineering. With the development of cutting edge MICP research, it is beneficial for this article to review the recent trend of MICP in construction engineering, so that a comprehensive understanding on microbial utilization for bio-cementation can be achieved.

## 1. Introduction

The construction industry contributes to massive economic and social development around the world. However, it has a broad carbon footprint due to high energy consumption, from the production phase of raw materials to the construction phase of structures. This accelerates global warming, and therefore the melting of icecaps and consequent rising of sea water levels. Such an environmental problem is affecting the quality of life of the world’s population. Concrete, cement mortar, and burnt clay brick have been identified as the building materials that emit carbon dioxide (CO_2_) in their manufacturing process. Cement functions as the conventional binding powder for the production of concrete and mortar. On the other hand, burnt clay bricks are commonly used to build masonry walls for buildings. Concrete is the second most widely used material on earth after water [1]. Around the world, cement production is increasing continuously, and it is known that around four billion tons of cement are generated per year [2]. Cement generation produced 6% of global anthropogenic carbon emissions, and the building construction and operation process has resulted in carbon emissions of 50% worldwide [3]. Producing each burnt clay brick (17 cm × 14 cm × 28 cm size) in a factory requires a consumption of 0.2 kg of coal for burning and a carbon emission of 1197.7 g of the burning process [4]. In addition, the demolition of old structures has led to an inadequate use of land for increasing construction waste [5]. These problems have triggered the need for a green solution to construction materials.

Bio-cementation is increasingly recognized as a green solution for the application of bonding in construction materials. Bio-cementation is a technique that uses microorganisms to produce calcium carbonate for construction purpose. Through microbiologically induced calcium carbonate precipitation (MICP), microorganisms can react with chemical components to produce minerals in the form of organic–inorganic compounds acting as a binding agent [6]. Numerous research articles have been published on the application of MICP to improve the mechanical properties of construction materials. Concrete fractures are detrimental to structural performance in terms of their service life [7]. This problem can be overcome by applying MICP of *Bacillus* bacteria to heal concrete cracks [8]. In particular, MICP has been used to repair concrete cracks [9,10,11,12], to reduce the porosity of concrete by pore clogging [13], to treat the surface of construction materials [14,15,16,17], to produce biological cement for sandstone bricks [4,17], to induce surface coating on concrete [18], and to stabilize dispersive soil [19].

A critical review of the MICP applications can deepen understanding of the role of bio-cementation in the construction of sustainable buildings. By assessing mechanical and chemical properties of bio-cemented construction materials, their robustness and durability can be characterized. The scientific evidence is able to pave a clear direction towards refined research on the mechanism of bio-cementation of microorganisms in the production of tangible bio-based construction materials that are comparable to conventional resources. As such, the objective of this article is to provide a detailed overview of bio-cementation in construction materials. In particular, the roles and the effectiveness of different species of microorganisms of inducing calcium carbonate precipitation for construction materials are overviewed.

## 2. Effects of Bacterial Treatment on Compressive Strength of Cement Composites

Based on evidence in the literature, MICP has been intensively explored using different species of microorganisms to improve the compressive strength of cement composites such as mortar and concrete. *Sporosarcina pasteurii* is the most studied species of *Bacillus* bacteria for MICP applications on cement composites. *S*. *pasteurii* are urease-producing bacteria that have the ability to induce sufficient calcium carbonate precipitation for bio-cementation to occur in concrete structures. The mechanism of bio-cementation of the bacteria is influenced by urea hydrolysis. The incorporation of nutrient medium extracted from urea yeast is necessary to keep *S*. *pasteurii* in an active metabolic state [20]. It should be noted that the nutrient medium resulted in a retarded cement hydration process, delayed hardening, and lower compressive strength of mortar at the early stage [21]. Jonkers and Schlangen [22] reported that mortar compression resistance could be reduced by 10% when adding bacteria and organic bio-mineral precursor compounds into cement pastes. However, bacterial mortar compression resistance improved during 28-days bio-calcification due to the significant precipitation of calcium carbonate by bacteria. The 28-day compressive strength of the bacterial mortar was found to be compatible with that of cement mortar. Lactose mother liquor, an industrial effluent from the dairy industry, is a good source of nutrients that can support the growth and urease activity of the bacteria [23]. In another study, it was reported that microbial mortars treated with calcium nitrate demonstrated compressive resistance twice as high as those of microbial mortars treated with calcium acetate and calcium chloride [24]. Interestingly, the generated bio-cement of native *Sporosarcina* sp. achieved 461% more unconfined compressive strength (UCS) and 120% more calcium carbonate content than the bio-cement generated by *S*. *pasteurii* DSMZ 33 [25]. These findings show that bio-calcification took time, because the activity of urease and carbonation processes of the bacteria required optimization for maximum precipitation of calcite to bind the materials.

The results of the tests of a published study indicated that the inclusion of *S*. *pasteurii* in a concentration of 10^5^ cells mL^−1^ enhanced the compressive strength of concrete by 22% (it increased from 24 to 28 MPa) [26]. However, improvement in the strength became less significant when the percentage of flying ash increased from 10 to 30% as a partial replacement of cement in concrete. Ameri et al. [27] reported a similar strength effect related to optimal bacterial concentration of 10^5^ mL^−1^ cells for self-compacting concrete with rice husk ash used as partial cement replacement. After the concrete cubes with 10% silica fume as a partial cement replacement were treated by *S*. *pasteurii*, the treated cement samples were tested to have an average 28-days compressive strength increase from 36 to 40 MPa at a bacteria concentration of 10^5^ cells mL^−1^ in comparison to the untreated ones [28]. Evidently, after an exposure to treatment by *S*. *pasteurii* at cell concentrations of 10^6^ cells mL^−1^, the compressive strength of lightweight aggregate concrete was noticed to improve by 20% [29]. For the purpose of making bio-mineralized mortars, lightweight expanded clay aggregates (LECAs) were first soaked in a bacterial suspension for six days before they were immersed in tap water for six days. The pre-wetted lightweight fine expanded shale aggregates acted effectively as internal nutrient reservoirs to provide delayed nutrient and water release supporting the metabolic state of microorganisms, and this led to improvement of the compressive strength of the bio-mineralized mortars [30]. Other than that, Feng et al. [31] found that MICP treatment by *S*. *pasteurii* could improve the properties of recycled fine aggregates (RFAs) of mortars due to adequate calcium carbonate precipitation on the mortar surfaces. The improved quality of RFA led to an increase in compressive strength of the mortars. It should be noted that sand stabilized with cement or geopolymer followed by MICP treatment led to a considerable increase in the compressive strength and stiffness due to the synergistic effects between chemical and microbial cementation [32].

*Bacillus* bacteria are recognized as Gram-positive bacteria that can induce a binder filling material (calcium carbonate (CaCO_3_) precipitation) to improve the strength and durability of concrete. It has been reported that cell walls of *Bacillus subtilis* are capable of mediating MICP, thus forming CaCO_3_ and resulting in a 15% improvement in concrete compression resistance [33]. On the other hand, it was observed that the dead and alive cells of *B*. *subtilis* had no positive effect on the compressive strengths of concrete. Mondal and Ghosh [34] investigated improvements of compression resistance of *B*. *subtilis*-treated concrete at three bacterial cell concentrations, namely, 10^3^ cells mL^−1^, 10^5^ cells mL^−1^, and 10^7^ cells mL^−1^. The highest strength improvement of the concrete was only recorded after treatment with a bacterial cell concentration of 10^5^ cells mL^−1^. However, a separate study revealed that the values of compressive strength of *B*. *subtilis*-treated lightweight aggregate concrete samples with and without steel fiber (at a bacterial cell concentration of 10^7^ cells mL^−1^) were slightly reduced by comparison to those of control concrete samples [35]. Another study indicated that concrete strength can be maximized with MICP based on an optimal concentration of *Bacillus megaterium* cells of 30 × 10^5^ CFU mL^−1^ [36]. The increase in compression resistance resulting from the treatment was observed to be more significant in concrete with a compressive strength of 50 MPa, and the value of increase in compressive strength of the treated concrete was noted as 25%. This is much higher than the 13% strength increment after MICP treatment of concrete with 30 MPa compressive strength. In a comparative experimental study of Nain et al. [37], concrete samples treated with *B*. *megaterium* showed a slightly higher compressive strength than those treated with *B*. *subtilis*.

It has been proven that curing conditions, such as temperature, relative humidity, wind speed, and sunlight exposure duration could affect the strength of bio-cemented mortars [38]. The bacteria used in the study were *B*. *subtilis*, which were then added directly to induce bio-cementation on cement mortars. The increase in temperature, relative humidity, and wind speed led to higher compressive strength, while the increase in exposure time to sunlight reduced the compressive strength of bio-cement mortars. Zhang et al. [39] have demonstrated that *Bacillus halodurans* improved the compressive strength of engineered cementitious composites. Shanmuga Priya et al. [9] reported improvements in the compressive strength of high-strength concrete (60 MPa) using *Bacillus sphaericus*. The inclusion of *Bacillus aerius* in concrete with 10% rice husk ash as partial cement replacement resulted in an improvement of compressive strength from 36 to 40 MPa [40]. Apart from this, fly ash concrete treated by *B*. *megaterium* demonstrated a strength improvement up to 40% with a fly ash content of 10% as partial replacement of cement [41]. *Bacillus pseudofirmus* was applied to modify recycled concrete aggregates using a bio-deposition method. The CO_2_ produced by the respiratory metabolism of *B*. *pseudofirmus* was used to produce calcium carbonate through MICP, leading to increased concrete compressive strength [42]. A significant improvement in concrete compressive strength was also observed when corn steep liquor was used as a growth medium for *Bacillus* sp. CT5 for concrete treatment [43]. A study was conducted on the treatment of concrete using the calcite-producing bacterial strain AKKR5 at a concentration of 10^5^ cells mL^−1^ [44]. The compressive strength of concrete with bacterial strain AKKR5 was found to increase by 11% at 28 days of age. However, the concrete compressive strength reduced as cement baghouse filter dust was utilized as a partial replacement of cement at 10, 20, and 30% in the concrete. These studies have shown that it is relevant to evaluate interactions among bacteria and nutrients, the source of calcium, and the environment in order to optimize the precipitation of calcium carbonate for the bio-cementation purposes of concrete.

The effect of bio-cementation on construction materials using bacteria other than *Bacillus* was also reported in several published studies. Ghosh et al. [45] noted that the compressive strength of cement–sand mortars could be increased by the addition of various concentrations of thermophilic anaerobic microorganism (*Shewanella* species) in mixing water. The highest increase of 25% in 28-day compressive strength of cement mortar was achieved with an addition of 10^5^ cell mL^−1^ bacterial concentration. The strength improvement was attributed to the growth of filling material in the form of calcium carbonate in the pores of the cement–sand matrix. Bio-OPC cement samples have been reported to be produced by deposition of bacterial calcium carbonate on cementitious materials [46]. In a study by Charpe et al. [46], a soil microbial solution with lentil seed powder (protein source) and sugar (carbon source) was used to replace water in the production of the bio-OPC cement samples. The average compressive strength of the bio-OPC cement samples increased by 23% compared to OPC cement samples. Bansal et al. [47] reported that halophilic bacteria *Exiguobacterium mexicanum* isolated from seawater could improve the compressive strength of concrete by 24%. Approximately a 10% increase in concrete compressive strength was observed with the incorporation of alkaliphilic/alkali-tolerant bacteria in silica fume concrete at 28 days [48]. Alkali-resistant bacteria in calcium formate medium was realized to improve the compressive strength of cement mortar at 28 days of curing [49]. Another important discovery revealed that effective microorganisms (EMs) consortia could be produced by combining three microorganisms, namely, lactic acid bacteria, photosynthetic bacteria, and yeast in a suitable liquid medium to formulate a binder. It was shown that the effective microorganisms could be used as a bio-superplasticizer [50] and could improve the compressive strength of concrete [51,52]. These findings demonstrated that a variety of microorganisms could be studied for the application of bio-cementation on concrete.

It can be summarized from Section 2 that urease activity, bacterial cell concentration, and the type and concentration of calcium source influenced the bio-cementation process of *Bacillus* bacteria in cement composites. In addition to bio-cementation, the robustness of the cement composites was further enhanced with the inclusion of raw materials such as steel fibers, rice husk ash, and silica fume. For microorganisms that do not produce urease, such as bacteria from the *Shewanella* species, the concentration of bacterial cells and the source of nutrients were found to have a significant influence on their calcium carbonate precipitation, resulting in filling and binding effects on the cement composites. However, further research is needed to examine other underlying factors that may induce bio-cementation on cement composites from the microorganisms.

## 3. Effect of Bacterial Treatment on Compressive Strength of Bricks

The production process of bio-bricks from MICP is more sustainable than the current processes of energy intensive production of bricks, such as kiln firing and the utilization of cement for manufacturing bricks. In the study of Bernardi et al. [17], bio-bricks were produced through MICP by adding *S*. *pasteurii* bacterial solution to sand by percolation. The percolation process was repeated before the molds were sealed to achieve full saturation for the bricks. The maximum compressive strength of the bio-bricks was discovered to be 2 MPa [17]. Using the same species of bacteria, Kumar et al. [53] created bio-bricks with a compressive strength of 4 MPa, and Lambert and Randall [54] produced bio-bricks with a compressive strength of 2.7 MPa. It should be noted that the bio-brick tested by Lambert and Randall [54] was made of calcium phosphate and a urea-rich solution produced by a urine stabilization process. Bu et al. [55] adopted a method of immersion in which the sand was placed in a rigid full-contact mold and then immersed in a medium of bio-cementation for seven days for the MICP to take place. The bio-brick gained a compressive strength of 1.3 MPa.

**Figure 1:**
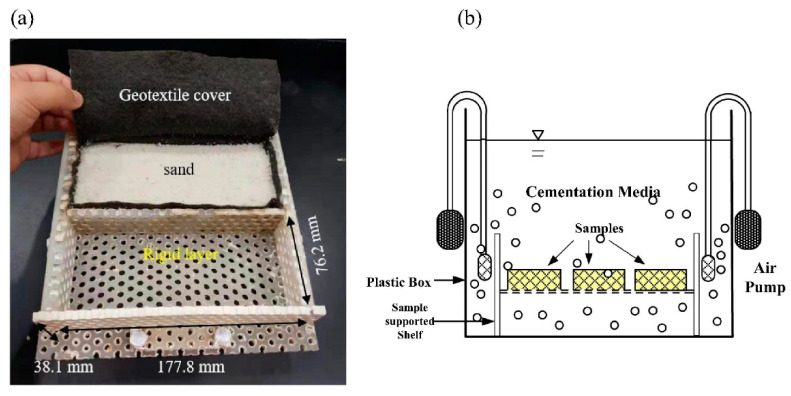
(**a**) Preparation of the bio-brick sample in a rigid mold in full contact. (**b**) Sketch drawing of batch reactor for immersion treatment method. Reprinted with permission from [58]. Copyright 2021 Elsevier.

Bio-brick compressive strength with *S*. *pasteurii* as a microbial agent has been improved with the inclusion of biopolymer (guar gum) [56], and the strength value could be further enhanced by increasing the number of treatments and the inclusion of fibers [57,58]. Figure 1 shows the preparation of a bio-brick in a full-contact rigid mold and a sketch drawing of a batch reactor for the immersing treatment method. Bio-bricks treated with a saturated condition of 50% were almost twice as strong as the samples treated with a saturated condition of 100% [4]. It was found that calcite precipitates were concentrated at the contact points of the particles, which led to a better improvement of resistance under the saturated condition of 50%. The compressive strength of bio-bricks with incorporated waste materials such as fly ash and rice husk ash [59], recycled concrete aggregates [60], and steel slag [61,62] were studied in depth. The bio-blocks using recycled concrete aggregates have achieved an average compressive strength of 4 MPa, and the strength value is compatible with that of natural aggregates [60]. It was noticed that the compressive strength of steel slag bricks after treatment with *Bacillus mucilaginous* bacteria through MICP ranged from 8 to 16 MPa, and the strength increased with an increased lime/steel slag ratio [61]. Carbonic anhydrase (CA) bacteria could be used to accelerate the carbonation process, which would solve the soundness problem and improve the mechanical properties and durability of steel slag bricks [62]. The surface treatment by MICP on cement-stabilized rammed earth blocks resulted in improved compressive strength from 12 to 15 MPa, and such indication implied a total increase in compressive strength of 25% [63]. These findings show that it is possible to optimize the process of MICP on bricks with different types of bacteria and incorporating different types of recycled products and wastes of raw materials.

It can be summarized from Section 3 that the compressive strength of bio-bricks depends on a number of factors. These factors include the type of microorganisms, the condition of MICP, the initial level of saturation, the type and quantity of supplementary or reinforcing raw material used, and the bio-brick production method. These factors influenced the strength optimization of the bio-bricks in such a way that their porosity was minimized, and simultaneously, the binding effect caused by the MICP was maximized. It was also observed that surface treatment by applying bacterial calcite precipitation could reduce the porosity of bricks, thereby improving their strength.

## 4. Fungal Treatment of Concrete

Fungi have great potential for the extraction or removal of heavy metals [64]. It has been shown that *Aspergillus niger* can effectively overcome the leaching of heavy metals from concrete incorporating waste foundry sand [65]. In addition, *Aspergillus* spp. isolates could be used to treat waste foundry sand [66]. Concrete with a 20% replacement of treated waste foundry sand demonstrated significant improvements in compressive strength, with an increase in the strength value from 23 to 33 MPa compared to that of untreated concrete. The strength improvement was attributed to the deposition of fungal spores in the pores of the cement–sand matrix, and this fact is supported by the reduction in concrete water absorption from 0.9 to 0.5%. Fang et al. [67] revealed that the fungus strain *Penicillium chrysogenum* CS1 increased the compressive strength of a sand column by 1.8 MPa in a fungal mineralization process. Based on the compressive strength achieved, it can be deduced that the fungus has a good potential to be used for the production of bricks.

Jin et al. [68] noted that various desirable properties of fungi could be used in the self-healing of concrete. Fungi can best adapt to deleterious environment such as low humidity, extreme temperatures, ultraviolet light, and high alkalinity that concrete could expose during its service life [68]. This capability implies that self-healing concrete mediated by fungi could possess long-term self-healing capacity. Fungal branching architecture could provide more nucleation sites and framework support for the calcium carbonate precipitates, thus enabling the fungal calcium carbonate to precipitate more effectively compared to yeast and bacteria. It has been suggested that fungi could seal wider cracks in comparison to the capability of bacteria in the bio-cementation process. To date, research on this subject is still in the initial stage, from which suitable strains of fungi for healing concrete cracks are being explored. Preliminary results have showed that fungal species with *Trichoderma reesei* spores had a great potential for healing cracks, because they have grown well in the hyphal mycelium with the precipitation of concrete crystals and calcites were clearly observed [69]. A study conducted by the same research group showed that *Aspergillus nidulans* could grow in concrete slabs surviving in a high pH environment caused by the leaching of calcium hydroxide from concrete, and this promoted the precipitation of calcium carbonate [70]. More research is needed to demonstrate the effectiveness of fungi in healing concrete cracks compared to existing bacteria reported in the literature.

**Figure 2:**
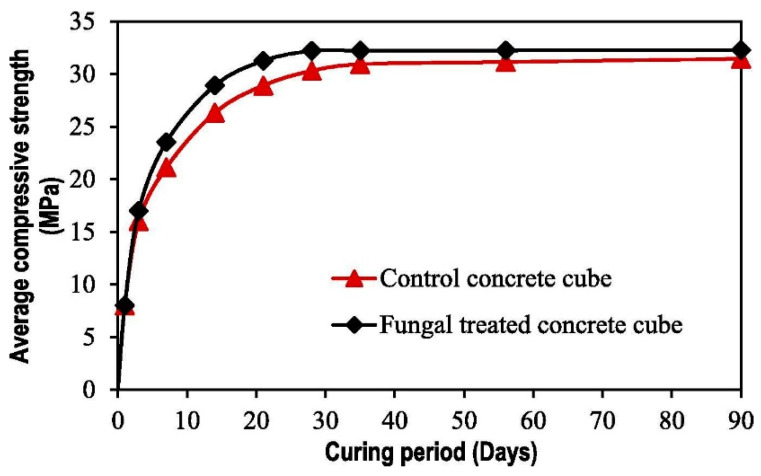
Development of average compressive strength with curing period for the control and concrete cubes treated with *C*. *ethanolica*. Reprinted with permission from [18]. Copyright 2021 Elsevier.

Recently, Wong et al. [18] found that fungal mineralization by *Candida ethanolica* could be used for the surface coating of concrete with polluted sand as a fine aggregate. At an initial optimal pH of 8.5, a calcium oxide concentration of 60 g L^−1^ and a concentration of fungal cells of 10^7^ cells mL^−1^, concrete cubes treated with the fungi were tested to have an average 28-day compressive strength of 32.2 MPa, which is 6.27% greater than that of the untreated concrete cubes (Figure 2). The chromium concentration in leachate of the treated concrete cube was reduced to below the World Health Organization (WHO) regulatory limit of 0.05 mg L^−1^ [65] compared to the heavy metal concentration of 0.22 mg L^−1^ in leachate of the untreated concrete cube. This demonstrated the efficiency of fungal mineralization to improve the strength and reduce the concentration of chromium in leachate of the fungal-treated concrete.

In summary, fungal mineralization has played an important role in the repair of cracks and surface treatment of construction materials. Through fungal mineralization, several fungal species were capable of precipitating calcium carbonate, which could improve the strength of construction materials. It was also shown that the mineralization of fungi was capable of encapsulating heavy metals, thus becoming a viable biotechnology for the treatment of contaminated sand. However, the affinity of fungi to trap the various types of heavy metal in fungal mineralization processes varies from one species to another. It is therefore recommended that future research should investigate the type and concentration of heavy metals that can be captured by various fungi species with the ability to induce calcium carbonate precipitation for the treatment of construction materials.

## 5. Water Absorption of Bio-Cemented Construction Materials

**Figure 3:**
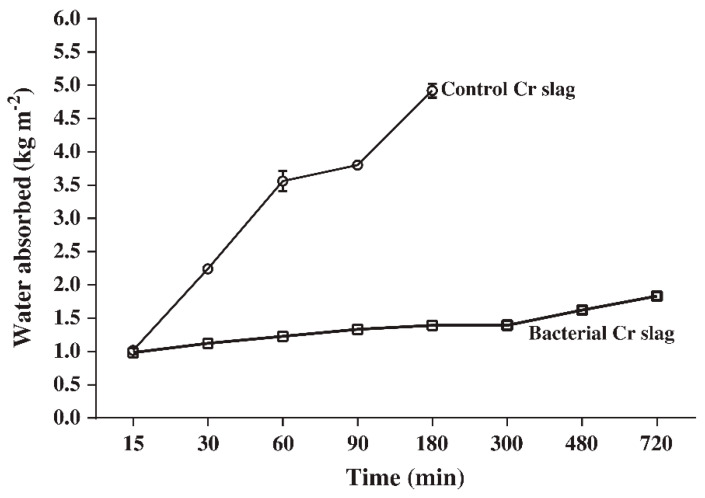
Water absorption of chromium slag bricks with bacterial treatment compared to control bricks at different time intervals (Note: Cr, chromium). Reprinted with permission from [71]. Copyright 2021 Elsevier.

In the study by Achal et al. [71], chromium slag was utilized to produce bricks with MICP application of ureolytic bacteria *Bacillus* sp. CS8 for surface treatment. The results of low water absorption of the treated bricks implied that they had a low permeability. It was also revealed that the chromium slag brick under bacterial treatment could absorb water four-fold less than that of the control brick without bacterial treatment, as shown in Figure 3. The study demonstrated that the layer of bacterial precipitated calcium carbonate on brick surfaces effectively reduced water absorption and permeability, thus improving their engineering performance to withstand the degradation process. It was observed that the chromium slag bricks treated with the bacteria were capable of resisting the effect of rain exposure and to develop high resistance against erosion as their leachability decreased [71].

**Figure 4:**
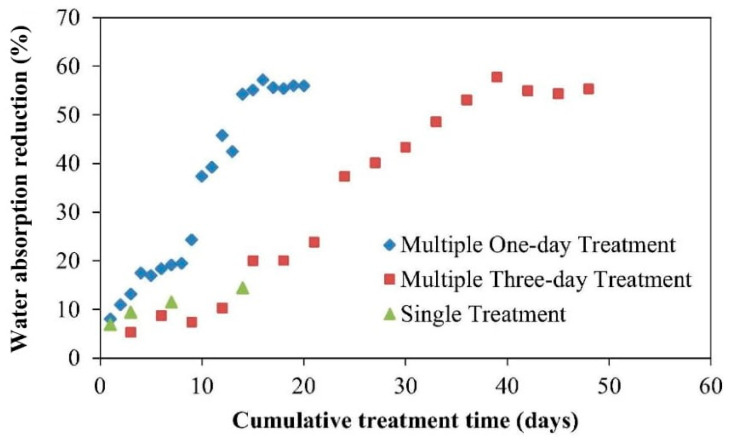
Reduction in water absorption in bricks with respect to different methods of treatment of CaCO_3_. Reprinted with permission from [72]. Copyright 2021 Elsevier.

Wang et al. [72] conducted research on CaCO_3_ for the repair of brick cracks and compared it to the similar brick application of using hydroxyapatite (HAP). The comparison was made to justify the advantage of HAP over CaCO_3_, which was known to be dissolved under low pH water solution. HAP was shown to have a lower solubility and dissolution rate compared to CaCO_3_. It has also been proven to have a higher resistance to acid attack by comparison to CaCO_3_. Results of water absorption for the CaCO_3_-treated bricks were obtained from tests based on a single treatment method, multi three-day treatment method, and multiple one-day treatment method (Figure 4). A maximum reduction of 14.40% in water absorption was recorded for the bricks exposed to the single-treatment method after a 14-day treatment, which was attributed to the lack of generation of phosphate or calcium ions for MICP. On the other hand, an 18% decrease in water absorption was observed when the bricks were subjected to four cycles of multiple treatments, which was significantly high compared to the single treatment. Finally, a 57% reduction in water absorption of the bricks was evident when they were subjected to 16 cycles of one-day treatment, which could be linked to the minimization of pores in bricks under this treatment. This finding provided a very useful insight into the most effective technique for CaCO_3_ precipitation that can be used in MICP to optimize the healing of brick cracks.

Raut et al. [73] studied bio-calcification technology to strengthen brick masonry using *S*. *pasteurii*. Water absorption was evaluated after the bio-calcification of brick masonry using optimized for urease production (OptU) media by close monitoring of up to 28 days. Water absorption tests were performed to measure the resistance of bio-calcified bricks to water penetration, which could affect their strength performance. The results of the study indicated that water absorption of the bio-calcified bricks with *S*. *pasteurii* and (OptU) media was reduced by 48.9%, and that an addition of nutrient broth (NB) in the media caused their water absorption to reduce by 19.95% compared to that of the control bricks. It can be interpreted from these results that the bio-calcification effect of *S*. *pasteurii* in OptU media was greater than that of *S*. *pasteurii* with NB. This could be related to the reduced water absorption of the bricks due to their increased durability and longevity after treatment with *S*. *pasteurii* in the OptU media.

**Figure 5:**
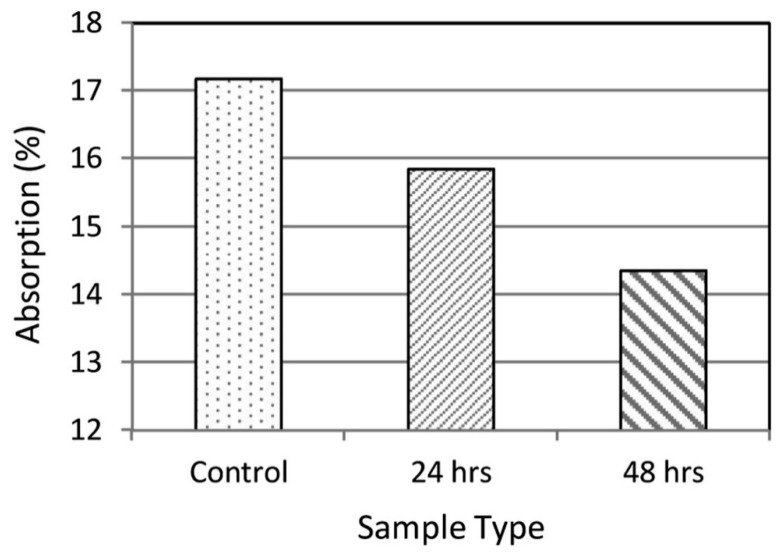
Water absorption of bacterial-treated concrete specimens for 24 and 48 h incubation periods, and control concrete specimen. Reprinted with permission from [74]. Copyright 2021 Elsevier.

Manzur et al. [74] investigated MICP to measure the efficacy of urease-positive bacteria to improve the water absorption performance of concrete. Periods of 24 and 48 h of bacterial incubation were selected for concrete treatment. Figure 5 shows the results of water absorption after the test of three concrete specimens, consisting of a control aggregate, treated brick aggregate at 24 h bacterial incubation, and treated brick aggregate at 48 h bacterial incubation. The results showed a reduction in water absorption of the concrete specimens due to MICP. Approximately 18 and 6% reductions in water absorption were observed in the results of the 48 and 24 h bacterial-treated concrete specimens, respectively. The massive reduction in water absorption was attributed to CaCO_3_ precipitation, which filled up the pore spaces of the brick aggregate and simultaneously worked as a particle binding agent in the concrete. Furthermore, it was realized that the longer bacterial incubation period could be related to the greater reduction in the water absorption due to the higher number of bacteria grown for calcium carbonate precipitation in concrete.

**Figure 6:**
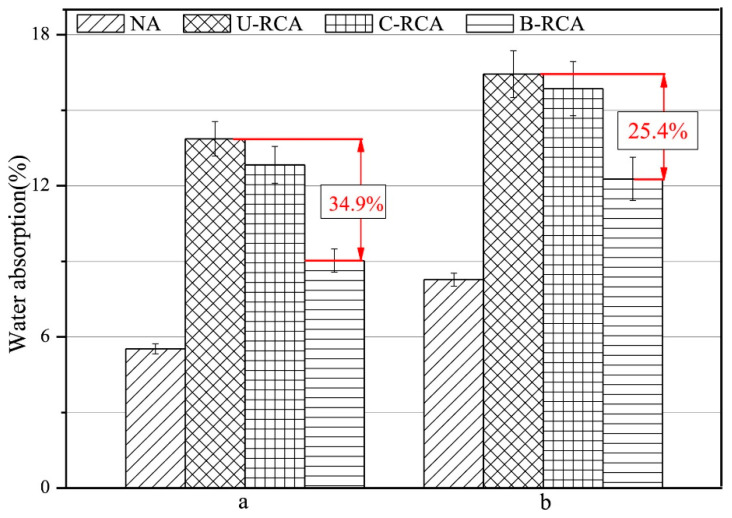
Water absorption of mortars that were prepared at a water to cement ratio of (**a**) 0.35 and (**b**) 0.5 (Note: NA, natural aggregate; U-RCA, untreated recycled concrete aggregate; C-RCA, recycled concrete aggregate treated without bacteria; B-RCA, recycled concrete aggregate treated with bacteria). Reprinted with permission from [42]. Copyright 2021 Elsevier.

Wu et al. [42] conducted research on recycled concrete aggregate (RCA) which had been improved by the deposition of calcium carbonate induced by *B*. *pseudofirmus* in the respiration process. This process differs from the conventional method of precipitation of CaCO_3_ using urea hydrolysis. The motivation of the RCA study was that it is beneficial to reuse demolished concrete from construction sector as mortar aggregate because disposal of the demolished concrete can add to the accumulation of waste in landfill, thus degrading the environment. Based on the results of the study, the water absorption values of untreated RCA (U-RCA) mortars were found to be higher at 12.3% for 0.5 water to cement (w/c) ratio, and 7% for 0.35 w/c ratio, respectively, compared to those prepared with the natural aggregate (NA), which were 8.3 and 5% at the respective water to cement ratios (Figure 6). It can also be seen in Figure 6 that mortars produced with bacteria-treated RCA (B-RCA) had a significant reduction in water absorption compared to those of U-RCA, which were 34.9% for 0.35 w/c ratio, and 25.4% for 0.5 w/c ratio, respectively. The apparent decrease in the water absorption of B-RCA indicated that deposition of calcite by the bacteria successfully clogged the pore spaces of the RCA, thereby decreasing the water uptake capacity of the aggregate. This improved the quality of the aggregate as a raw material for mortar production.

It can be summarized from Section 5 that water absorption is an important parameter for measuring the durability of construction materials. It is known that the durability of a construction material increases with a decrease in water absorption due to its dense microstructure and low porosity. Several studies have found that the water absorption of bacterial-treated concrete decreased due to the close packaging of particles, as reflected by its microstructure that could be attributed to the presence of calcium carbonate in voids. However, it should be pointed out that the percentage reduction in water absorption from concrete varied from one mixing design to another, as well as depending on the methods of bacterial treatment. It is clear from the results of the literature that the addition of materials such as pozzolana and waste aggregate reduced the water absorption of bacterial-treated concrete due to the refinement of pore spaces in the concrete, which effectively decreased its seepage of water. Gabriec et al. [75] noted that the reduction in water absorption was more significant in concrete with fine fractions and concrete with aggregates from low quality concrete.

## 6. Morphological and Chemical Evidence of Bio-Cemented Construction Materials

Literature studies on the morphology of scanning electron micrographs (SEMs) of bio-cemented construction materials provided an in-depth understanding on the binding mechanism of the particles within the materials. On the other hand, a review of chemical compositions of bio-cemented construction materials provided information on the mechanism of MICP to solidify them. In this regard, literature discoveries from X-ray diffraction (XRD) and energy dispersive X-ray (EDX) of materials are important for providing evidence on the chemical constituents that characterize their bio-cementation process. Detailed analyses and discussions of the literature related to these aspects are presented in this section.

### 6.1. Microstructures and Chemical Characterization of Bio-Cemented Sand Materials and Mortars

**Figure 7:**
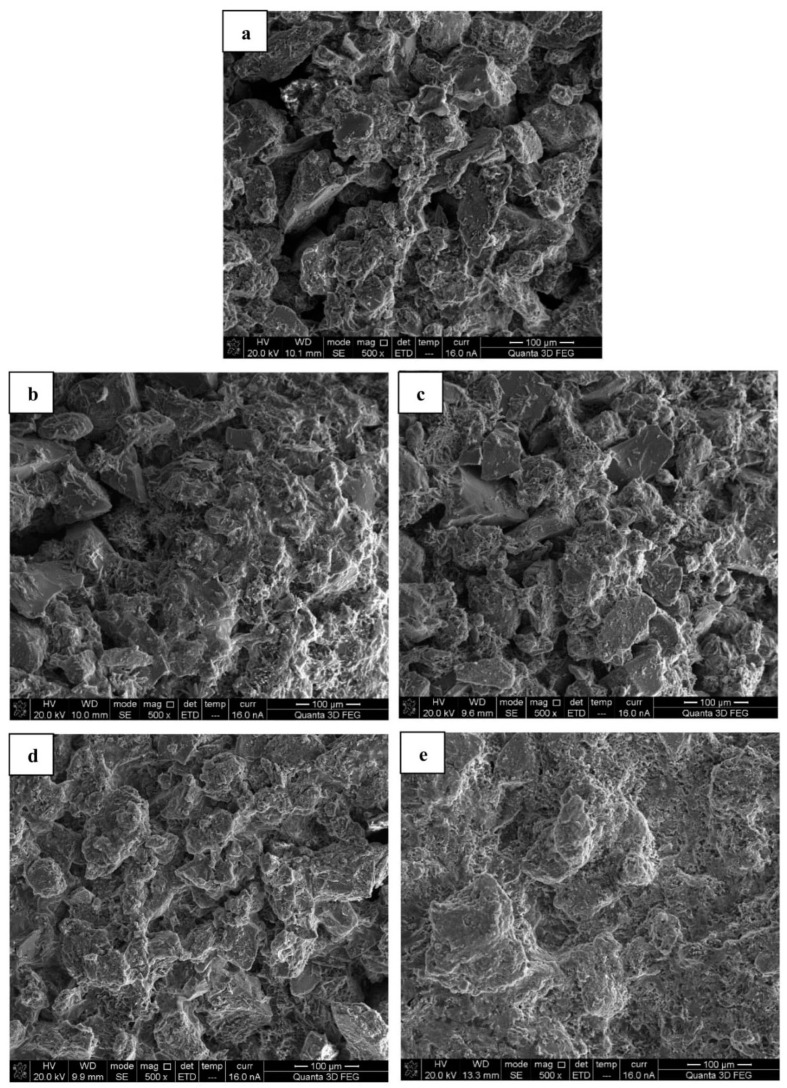
Scanning electron micrographs of (**a**) sand particles, and bio-cemented sand materials with (**b**) one spray; (**c**) three sprays; (**d**) five sprays; and (**e**) seven sprays. Reprinted with permission from [76]. Copyright 2021 Elsevier.

**Figure 8:**
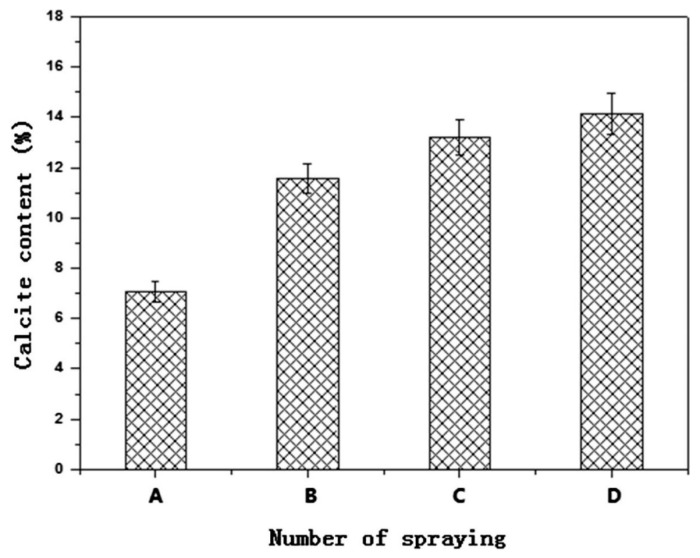
Effect of number of spraying on calcite content of bio-cemented sand material samples (Note: A, spraying once; B, spraying three times; C, spraying five times; D, spraying seven times). Reprinted with permission from [76]. Copyright 2021 Elsevier.

Zhan and Qian [76] researched binding mechanisms of sand particles using MICP based on *Paenibacillus* bacteria. In their work, the binding effect of sand particles with bio-cement from the bacteria was evaluated based on the number of times the bio-cement was sprayed on the sand particles. For the bio-cementation of the sand particles, the spraying process was implemented up to seven times. The mechanical properties of bio-cemented sand materials improved with an increase in the number of spraying time, because microstructures of the materials became denser as a result of the bio-cementation process which effectively clogged their void spaces. Figure 7 shows the microstructures of the sand before and after the bio-cementation process. In Figure 7a, it is seen the sand particles were loosely distributed with significant pore spaces in between. However, the microstructures of the bio-cemented sand materials became denser and closer in packing of particles after exposure to one, three, and five sprays of the bio-cement (Figure 7b–d). After an exposure to seven sprays of the bio-cement, the microstructure of the bio-cemented sand material was found to be the densest and the particles were most closely packed by comparison to all microstructures of the bio-cemented sand materials observed under the study. This could be related to the increase in calcium carbonate content and the reduction in average porosity of the bio-cemented sand material with each increase in the number of sprays of the bio-cement. Zhan and Qian [76] reported that by increasing the number of bio-cement sprays, the average calcium carbonate content and average porosity of the bio-cemented sand materials were found to increase from 7.08% (one spray) to 14.36% (seven sprays); and to reduce from 18.3% (one spray) to 13.3% (seven sprays), respectively. To quantify calcium carbonate of the bio-cemented sand material, the method of acid washing was applied. A solution of hydrochloric acid was used to dissolve the calcium carbonate from samples of the bio-cemented material. The average calcium carbonate quantities for the samples with bio-cement spraying once, three times, five times, and seven times were found to be 7.08, 11.56, 13.22, and 14.36%, respectively (Figure 8). The highest quantity of calcium carbonate precipitated on the bio-cemented sand material of 14.36% after seven sprays supported the microstructural finding that the pore spaces of the material were effectively filled up to yield its densest condition.

**Figure 9:**
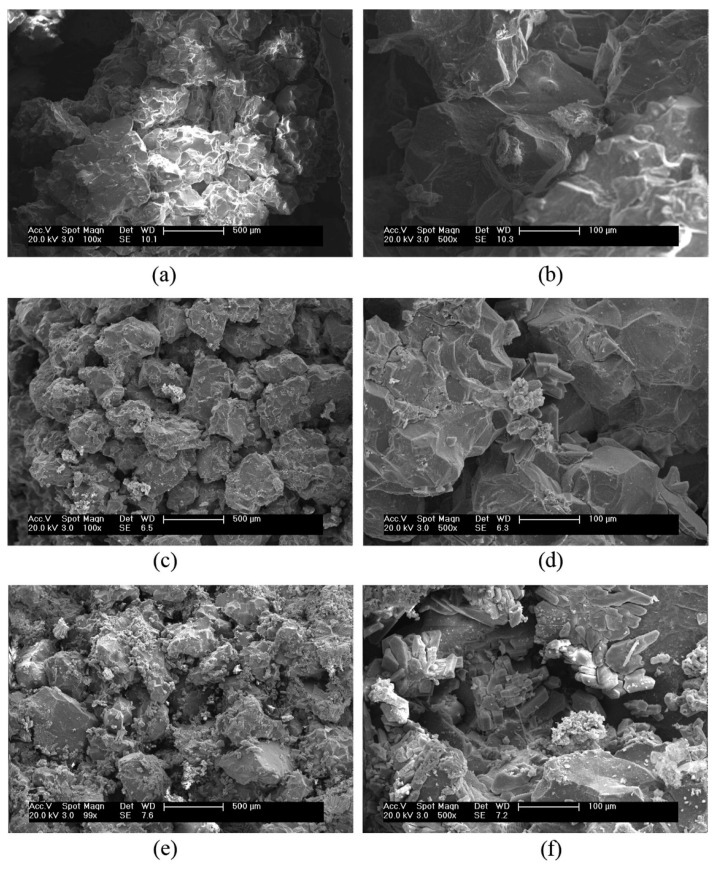
Microstructures of bio-sandstone improved with the bio-composite cement after (**a**,**b**) two injections; (**c**,**d**) four injections; and (**e**,**f**) six injections. Reprinted with permission from [77]. Copyright 2021 Elsevier.

A similar trend could be observed in the microstructures of bio-sandstone improved with up to six injections of bio-composite cement in the published work of Yu et al. [77]. *S*. *pasteurii* was used to produce bio-composite cement with an ultimate aim of enhancing the mechanical properties of the bio-sandstone through injections. Microstructures of the bio-sandstone, which have been subjected to different numbers of bio-composite cement injections, are shown in Figure 9. The morphology of bio-composite cement was characterized by irregular flakes, as indicated in Figure 9b,d,f. It is also evident that the microstructure of the bio-sandstone became denser as the number of injections increased. After six injections of the bio-composite cement (Figure 9e,f), microstructures of the bio-sandstone were the densest among all the microstructures seen in Figure 9. This has been attributed to the reduction in porosity in the bio-sandstone as a result of calcium carbonate precipitation induced by the bacteria from the injections. Bacterial calcium carbonate acted as a bonding agent that sealed the pore spaces of the bio-sandstone. A limitation of the study is that an XRD test was not performed to quantify the amount of calcium carbonate in bio-sandstone treated with bio-composite cement. Despite the positive result, more research should be conducted on the effect of bio-composite cement injected more than six times on bio-sandstone microstructures to ensure that the optimization of bio-cementation was achieved.

**Figure 10:**
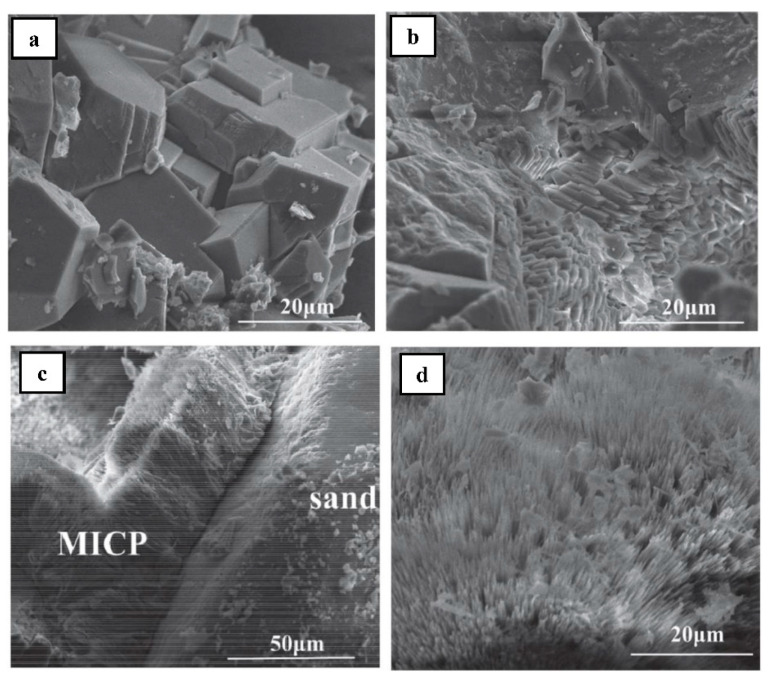
Microstructures of microbial mortars treated with three types of calcium source and three pumped batches: (**a**) chloride sample; (**b**) nitrate sample; (**c**,**d**) acetate sample (Note: MICP, microbiologically induced calcium carbonate precipitation). Reprinted with permission from [24]. Copyright 2021 Elsevier.

**Figure 11:**
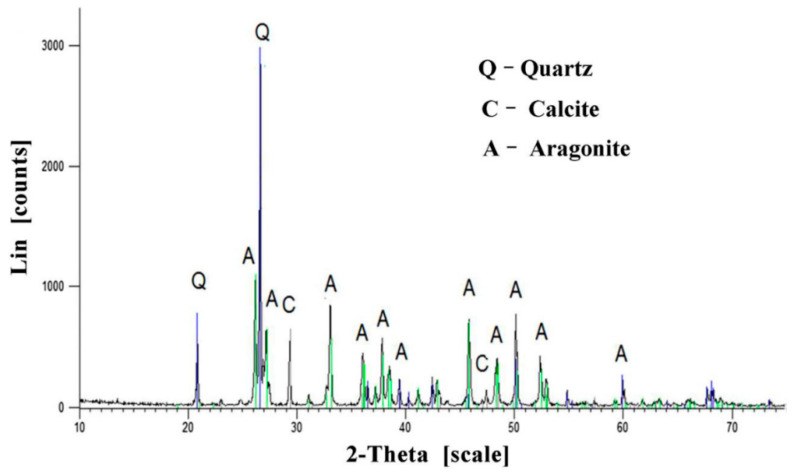
XRD result of microbial mortar treated with calcium acetate. Reprinted with permission from [24]. Copyright 2021 Elsevier.

According to Zhang et al. [24], the effect of MICP on microbial mortar microstructures depends on the type of calcium source used for bio-cementation. Three types of calcium sources were used for the bio-cementation of microbial mortars induced by *S*. *pasteurii*. These were calcium acetate, calcium nitrate, and calcium chloride. The microbial mortars were produced using grouting technology of pumping three batches of bacterial solution (*S*. *pasteurii* and culture medium), fixative solution (50 mM calcium source), and nutrient solution (a mixture of calcium source and urea with equal molar concentrations of 0.5 M). Scanning electron micrographs of microbial mortars with all three types of calcium source are depicted in Figure 10. It is noted in Figure 10a that the chloride sample had a smooth surface and large hexahedral calcium carbonate crystals. On the other hand, Figure 10b shows that the nitrate sample had calcium carbonate crystals which are small and of hexahedron shape. These hexahedral calcium carbonate crystals are most probably calcite [24]. Needle-shaped calcium carbonate crystals were seen in the micrographs of the acetate sample (Figure 10c,d). The needle-shaped morphology of crystals is typically characterized by aragonite, which is one of the three polymorphs of calcium carbonate. The other two polymorphs of calcium carbonate are vaterite and calcite. It is important to note that calcium carbonate can crystallize in any of three different crystal phases or “polymorphs”: calcite, aragonite, or vaterite, all with the same structural formula of CaCO_3_ [78]. The XRD result of the acetate sample is presented in Figure 11. It shows that calcium carbonate in the acetate sample was formed by 88% aragonite and 12% calcite with the peak diffraction of aragonite reaching a maximum of 2*θ* = 26.223°. This confirmed the presence of aragonite as the predominant calcium carbonate crystals in micrographs of the acetate sample in Figure 10c,d.

### 6.2. Microstructure and X-Ray Diffraction Evidence of Bio-Brick

**Figure 12:**
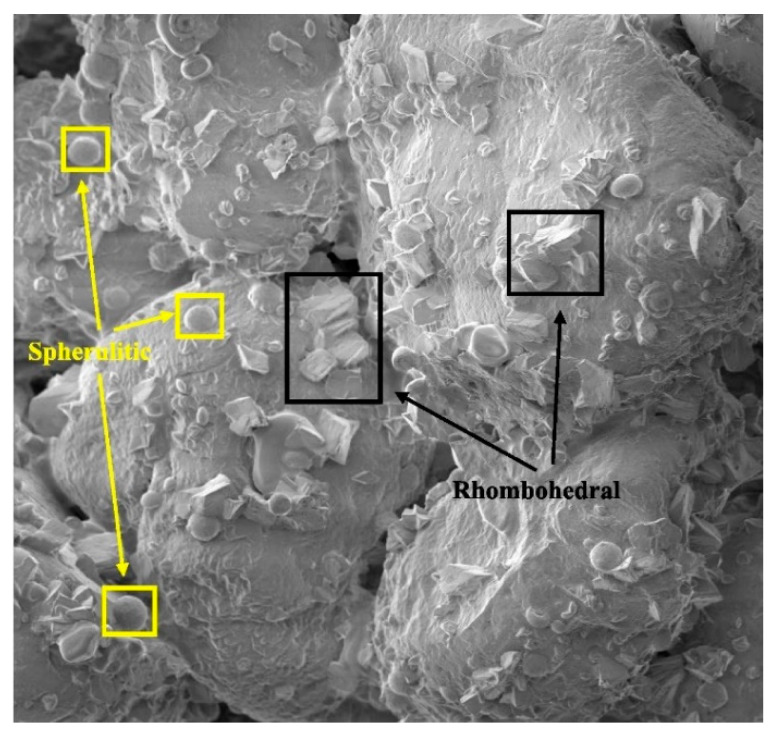
Scanning electron micrograph of bio-brick treated by MICP. Reprinted with permission from [57]. Copyright 2021 Elsevier.

**Figure 13:**
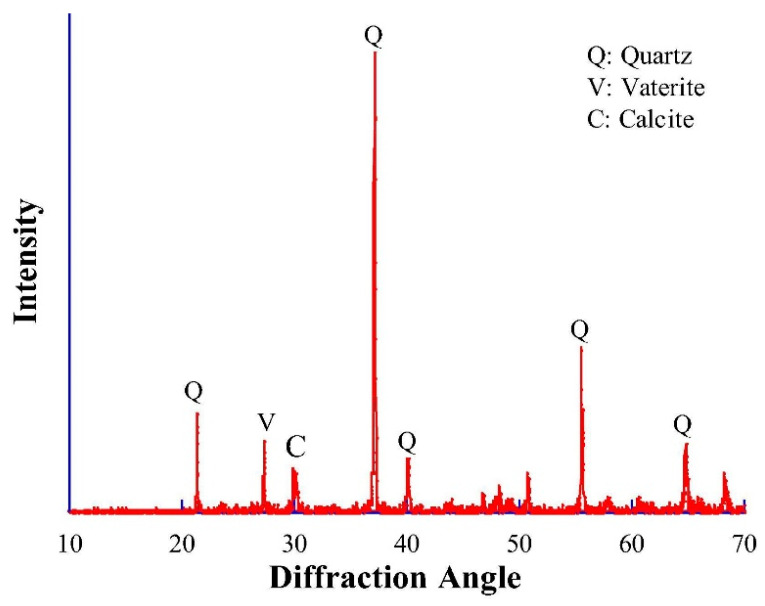
XRD result of bio-brick treated by MICP. Reprinted with permission from [57]. Copyright 2021 Elsevier.

Further microstructural evidence on bio-cementation can be observed in the micrograph of MICP-treated bio-bricks shown in Figure 12, with reference to the study of Li et al. [57]. In their study, bio-bricks were produced from materials including fibers, sand, and clay with the application of multiple MICP treatments. It was observed from the micrograph that there were distinctive images of spherulitic and rhombohedral crystals that meant the existence of vaterite and calcite. The XRD results of the bio-brick shown in Figure 13 confirmed the existence of the two types of mineral produced from MICP. Apart from quartz (the predominant mineral of sand), high diffraction peaks of vaterite and calcite at diffraction angles of 27.1° and 30.1°, respectively, were observed in the XRD result of the bio-brick. This discovery helped to better understand that fibers can be effectively applied with MICP to reinforce and strengthen bio-bricks.

### 6.3. Microstructures and Chemical Evidence of Concrete Treated with Microorganisms 

**Figure 14:**
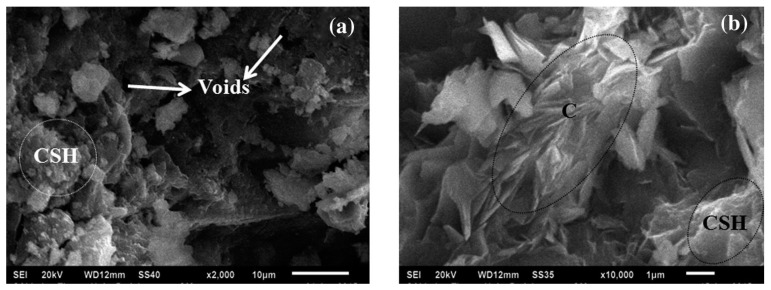
Micrograph indicating: (**a**) silica fume concrete (SF10) and (**b**) bacterial silica fume concrete (BSF10) (Note: CSH, calcium silicate hydrate; C, calcite). Reprinted with permission from [48]. Copyright 2021 Elsevier.

**Figure 15:**
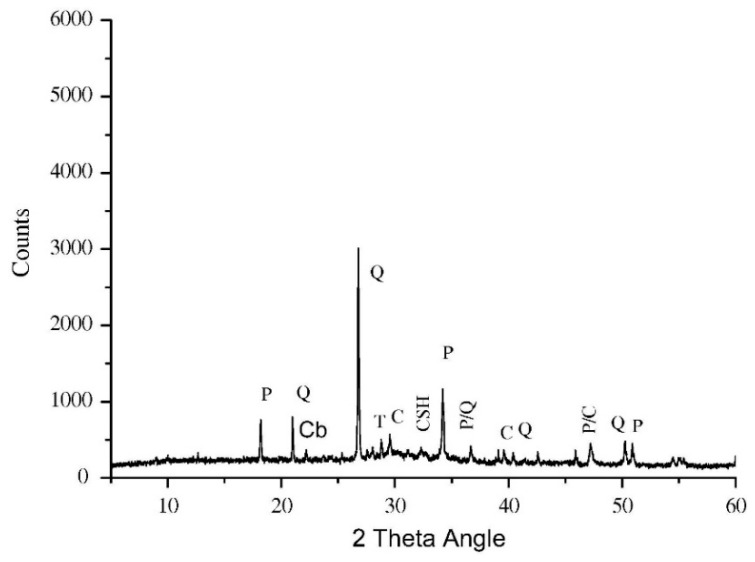
XRD result of bacterial silica fume concrete (BSF10) in 28-day curing (Note: P, Portlandite; Q, quartz; C, calcite; CS, calcium silicate; CSH, calcium silicate hydrate; Cb, Cristobalite). Reprinted with permission from [48]. Copyright 2021 Elsevier.

Microstructural evidence of bacterial silica fume concrete compared to that of silica fume concrete from the published work of Siddique et al. [48] is presented in Figure 14. The silica fume concrete had 10% silica fume as a partial replacement of cement and was treated with a bacterial culture of 10^5^ CFU mL^−1^ of water under MICP. Without bacterial treatment, the morphology of the silica fume concrete was predominated by calcium silicate hydrate (CSH) crystals which significantly reduced the pores of the concrete (Figure 14a). The CSH crystals are the bonding agents of cement hydrolysis in concrete. The fineness of the silica fume also contributed to the reduction in concrete pores. A denser microstructure can be seen in the micrograph of the bacterial silica fume concrete, as shown in Figure 14b. The particles present in the micrograph of the figure were tightly packed relative to each other, thereby minimizing the concrete voids. This was largely attributed to the precipitation of calcium carbonate on the surface of the bacterial silica fume concrete that further reduced the concrete pores and increased its robustness. In this respect, bacterial calcium carbonate worked as a filler of the bacterial silica fume concrete. The XRD result of the bacterial silica fume concrete is indicated in Figure 15. It can be seen in the figure that there were significant diffraction peaks of Portlandite, quartz, calcite, calcium silicate, calcium silicate hydrate, and cristobalite. The presence of a calcite crystalline phase in the XRD result confirmed that MICP occurred on the surface of the bacterial silica fume concrete.

**Figure 16:**
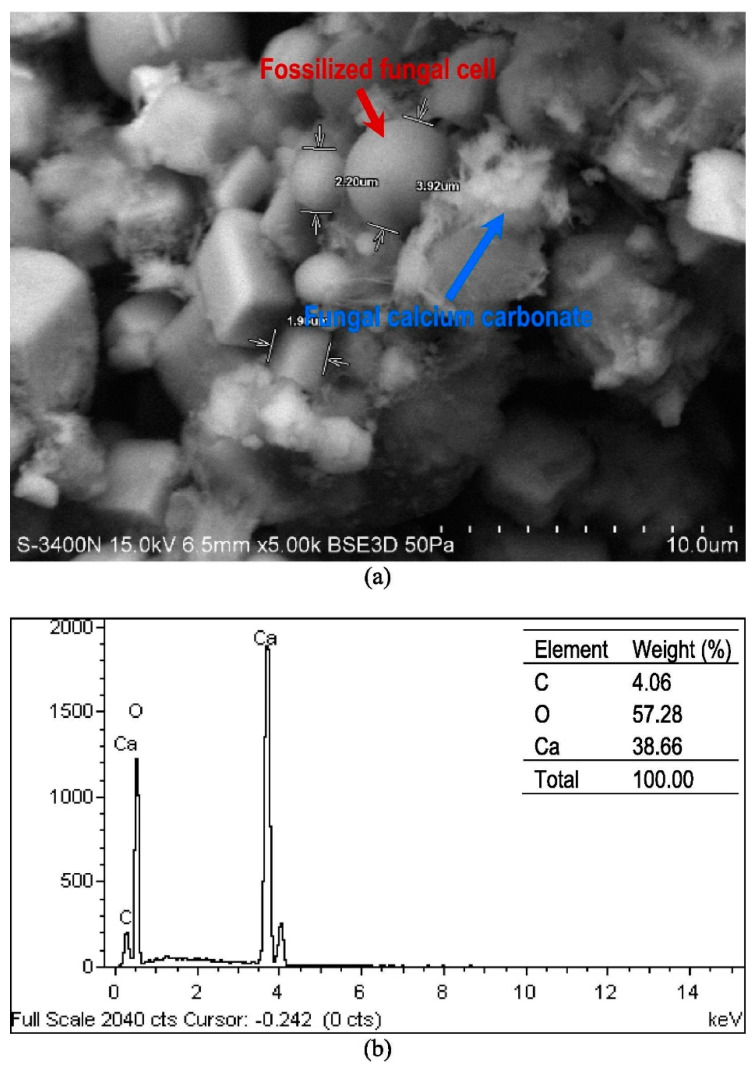
(**a**) Scanning electron micrograph (SEM) and (**b**) energy dispersive X-ray (EDX) result of *C*. *ethanolica* treated concrete cube specimen (Note: C, carbon; O, oxide; Ca, calcium). Reprinted with permission from [18]. Copyright 2021 Elsevier.

The calcium carbonate mineral could also be identified by the morphological result of fungal surface treatment of concrete cube by *C*. *ethanolica* from the study of Wong et al. [18]. A fossilized cell and a CaCO_3_ crystal due to the fungal surface treatment of the concrete cube could be traced in a scanning electron micrograph of Figure 16a. The microstructure of the fungal treated concrete cube showed that its pores were clogged with CaCO_3_ crystals. The dense morphology of the fungal treated concrete cube proved that deposition of calcium carbonate due to MICP was effective in minimizing its porosity. The presence of calcium carbonate mineral in the treated concrete cube was verified by the energy dispersive X-ray (EDX) result as indicated in Figure 16b. High elemental peaks of carbon (C), oxide (O), and calcium (Ca) were detected in Figure 16a. These elements were necessary for the formation of the calcium carbonate mineral which acted as a protective layer of the surface of the concrete cube.

After reviewing Section 6, it can be confirmed that the precipitation of calcium carbonate induced by microorganisms improved the proximity in the packaging arrangement of bio-cemented construction materials, as observed in their scanning electron micrographs. This was mainly attributed to the action of the microbial-induced calcium carbonate crystals that created clogging and mineralization impacts to minimize void spaces of the bio-cemented construction materials. The XRD results of the bio-cemented construction materials showed obvious diffraction peaks of calcite, aragonite, vaterite, and quartz. This provided chemical evidence on the binding effect caused by the microorganisms on the sand particles of the bio-cemented construction materials.

## 7. Application of Bio-Cementation in Construction Practice

In construction practice, Lors et al. [79] found that *B*. *pseudofirmus* could induce calcium carbonate precipitation, which offered an eco-friendly solution to repair concrete wall micro-cracks of a nuclear structure instrumented under a pressure of 500 kPa. It must be noted that the ability of bacteria to survive in a highly alkaline concrete environment during the formation of calcium carbonate crystals in concrete implies that the presence of the bacteria did not have a negative impact on the hydration reaction [80]. The micro-cracks of the concrete walls were sealed with calcium carbonate by spraying a bacterial suspension of bio-precipitation on the concrete walls. The repaired surfaces of the concrete walls were observed for minimal deformation due to exposure to high pressure. Further laboratory findings from the work of Lors et al. [79] revealed that precipitated calcium carbonate in the micro-cracks of the concrete could sustain an air flow at a pressure of 450 kPa. This showed that calcium carbonate induced by the bacteria was highly pressure-resistant as well as being chemically compatible with concrete in a highly alkaline environment. In summary, it can be affirmed that the method of spraying bio-precipitation is viable for the repair and sustainable rehabilitation of concrete structures.

## 8. Conclusions

It can be concluded from the critical review of the articles that microorganisms have played an important role in bio-cementation on construction materials including concrete, brick, and mortar. The effectiveness of microorganisms in inducing bio-cementation depends to a large extent on the microbial type, as well as the optimal conditions of MICP. *S*. *pasteurii* has been identified as the most studied microorganism for MICP on construction materials due to its high urease activity, growth rate, and reactivity to a source of calcium. The calcium carbonate precipitated by MICP served as a filler and cementing agent that minimized the porosity of construction materials. In addition to MICP, the inclusion of additional materials such as rice husk ash, silica fume, fly ash, and chromium slag as partial cement substitutes in construction materials could further reduce their porosity. It was the reduction in porosity of construction materials by MICP that led to the improvement of their mechanical properties. In this regard, MICP resulted in enhanced compressive strength and reduced water absorption of construction materials through crack repair, surface treatment, and bonding effects. Scanning electron micrographs and XRD results of bio-cemented construction materials from the literature studies revealed the presence of calcite, which is the most stable calcium carbonate mineral precipitated by microorganisms in the MICP. Despite the positive results in the literature, further research should be carried out to investigate the fire resistance of bio-cemented construction materials in order to establish a complete understanding of their fire durability. With the advancement of the research, the prospect of commercializing bio-cementation in construction materials seems promising.

## Data Availability

No new data were created or analyzed in this study. Data sharing is not applicable to this article.
